# Inferring influence of people's emotions at court on defendant's emotions using a prediction model

**DOI:** 10.3389/fpsyg.2023.1131724

**Published:** 2023-03-06

**Authors:** Yun Song, Tianyi Zhao

**Affiliations:** ^1^Rule of Law Institute, Northwest University of Political Science and Law, Xi'an, China; ^2^School of Health and Medicine, Harbin Institute of Technology, Harbin, China

**Keywords:** emotion prediction, deep learning, judgement, AI in law, emotion at court

## Abstract

People's emotions may be affected by the sound environment in court. A courtroom's sound environment usually consists of the people's voices, such as the judge's voice, the plaintiff's voice, and the defendant's voice. The judge, plaintiff, and defendant usually express their emotions through their voices. Human communication is heavily reliant on emotions. Emotions may also reflect a person's condition. Therefore, People's emotions at the Court must be recognized, especially for vulnerable groups, and the impact of the sound on the defendant's motions and judgment must be inferred. However, people's emotions are difficult to recognize in a courtroom. In addition, as far as we know, no existing study deals with the impact of sound on people in court. Based on sound perception, we develop a deep neural network-based model to infer people's emotions in our previous work. In the proposed model, we use the convolutional neural network and long short-term memory network to obtain features from speech signals and apply a dense neural network to infer people's emotions. Applying the model for emotion prediction based on sound at court, we explore the impact of sound at court on the defendant. Using the voice data collected from fifty trail records, we demonstrate that the voice of the judge can affect the defendant's emotions. Angry, neutrality and fear are the top three emotions of the defendant in court. In particular, the judge's voice expressing anger usually induces fear in the defendant. The plaintiff's angry voice may not have a substantial impact on the defendant's emotions.

## 1. Introduction

A range of disciplines are interested in emotion perception, including psychology, psychiatry, and social neuroscience. Especially emotions can affect the way a person thinks and makes decisions. A person's decision making may be influenced by his emotions or those of other people. Therefore, it is a key question to examine the defendant's emotions in court. According to existing research, court judgment might be affected by the emotions of people at court (Nunez et al., 2016). The study of the relationship between a defendant's emotions at trial can explore two key challenges. Does the defendant's emotion affect the court's judgment? What can we do to avoid bias caused by people's emotions at court, such as plaintiffs, judges, and defendants? Unbiased judgments and decision-making are especially important for defendants, as they are a vulnerable group in court.

Research has shown that emotions are influenced by sound environments (Meng et al., 2020). Few studies have investigated the impact of the sound environment at court on the defendant's emotions. It is primarily people's voices that create the sound environment in court, such as the judge's voice and the defendant's voice. By analyzing the impact of the courtroom acoustic environment on the defendant's psychological state, we can analyze the judge's voice and the defendant's voice. In addition, we can also infer from the emotions of different characters in court to determine whether their voices also have an impact on the defendant. Emotions can affect humans to a great extent (Clore and Huntsinger, 2007).

To analyze the influence of the listener's voice on its emotion, one of the important problems is to identify a person's emotion from the speech signal. In the court, the correct identification of people's emotions is the basis for the study of the judge's emotion and the relationship between the judge's judgment and decision. For example, the pain and fear of victims has a lot to do with the harm they have suffered. In court, when someone gives false evidence, he or she gets scared. Cognition of human emotion can be carried out through various means such as face, touch, vision, questionnaire and sound (Schirmer and Adolphs, 2017). However, most of them are absent in specific cases. Although it is necessary to study the relationship between the emotions of witnesses and court decisions, it is not easy to understand the emotions of witnesses in a timely manner. This paper investigates a speech-based deep learning approach to predict human emotion.

Due to its important position in psychology, psychiatry, social neurology and many other disciplines, speech recognition has become a hot spot in current artificial intelligence research. There are methods for emotion recognition based on sounds. Voice-based emotion recognition generally includes the following steps (Koolagudi and Rao, 2012). The first step is feature representation. The second step is the classification of sentiment. In the recognition process, feature extraction is an important link to make up for subjective emotions and digital signals. At present, some artificially designed features for speech recognition, such as: motivating factor, prosody factor, voice pulling factor and other mixed features. To identify different emotion types, linear and nonlinear machine learning algorithms are a commonly used identification method. So far, the classification methods of speech emotion recognition are: the Maximum Likelihood Principle (MLP), Bayesian Networks (BN), Hidden Markov Model (HMM), Support Vector Machine (SVM), Gaussian Mixture Model (GMM), and so on (Khalil et al., 2019). In speech emotion recognition, feature extraction is very meaningful. Although researchers have put a lot of effort into manual design, the characteristics of manual design are often some low-level. These characteristics may not be enough to discern emotions. How to automatically extract speech features with higher-level features is an urgent problem to be solved. Due to the successful application of deep learning technology in image analysis, natural language processing and other fields, it provides a feasible way to realize automatic identification of this problem. Compared with the traditional algorithm, the algorithm has great advantages in complex structure, feature discovery, noise removal and so on.

Although multi-layer speech recognition methods such as one-layer, two-layer convolutional neural network and LSTM have been widely used, they all belong to shallow layers. Compared with the shallow mode, the deep multi-layer convolution mode can learn better representations, because deep neural network can extract the complicated signals included in the sound. Based on LSTM convolutional neural network, a deep neural network-based feature learning model based on LSTM is established (Lim et al., 2016). Researchers combined principal component analysis with deep convolutional neural networks and extracted speech signal characteristics by combining linear and nonlinear modes (Barros et al., 2015). Zilakis et al., al suggest utilizing Convolutional Neural Networks and 50-level ResNet to identify different emotions (Badshah et al., 2017). Tang et al. combines ResNet with Convolutional Neural Networks to achieve significant improvement (Tang et al., 2018). At present, there are many emotion recognition methods based only on speech signals. However, current models are not well suited for emotion recognition in courtrooms. Generally, existing methods have the following shortcomings: (1) Although there are many methods of integrating models, such as convolutional neural network, LSTM, etc., the design of these models is not perfect, and it is difficult to effectively integrate various models; (2) The existing research has not considered that different contributions of the different parts of the speech signal are different during emotion recognition. In order to improve the overall performance of the system, we proposed a method to organically combine multiple neural network modes (Song and Wei, 2021). The proposed method incorporates convolutional networks and LSTM. On this basis, an attention-based model is built and incorporated into the system.

Using ResCNN-SER, we are able to analyze voices in the courtroom, especially judges and plaintiffs, to inference the emotions of defendants. No datasets are currently available. So we collected 50 trial records. On this basis, the ResCNN-SER method is used to study the influence of court sound environment on the defendant's emotions. Using the proposed method, we can study how the courtroom sound environment affects the defendant's emotions. The study found that the psychological states of accused criminal suspects mainly include fear, anger and neutrality. Defendants tend to be frightened when judges get angry. The difference is that the plaintiff's venting of anger has little effect on the defendant's emotions.

## 2. Method

There are two contents in this paper: (1) We apply the method proposed in our previous work for emotion recognition; (2) The influence of court sound environment is analyzed. First, using convolutional neural network, LSTM and attention mechanism, the original features and time-critical features are captured and analyzed. In Section 2, we produced a profile of 50 court records. In view of the fact that the acoustic environment of the court is dominated by the voice of the people, for the defendant, we use the voice of the judge and the plaintiff as the acoustic environment. Using the method of this study, we can analyze the impact of the judge's conversation with the plaintiff on the defendant's emotion.

### 2.1. Introduction to the method used for emotion recognition

**Figure 1:**
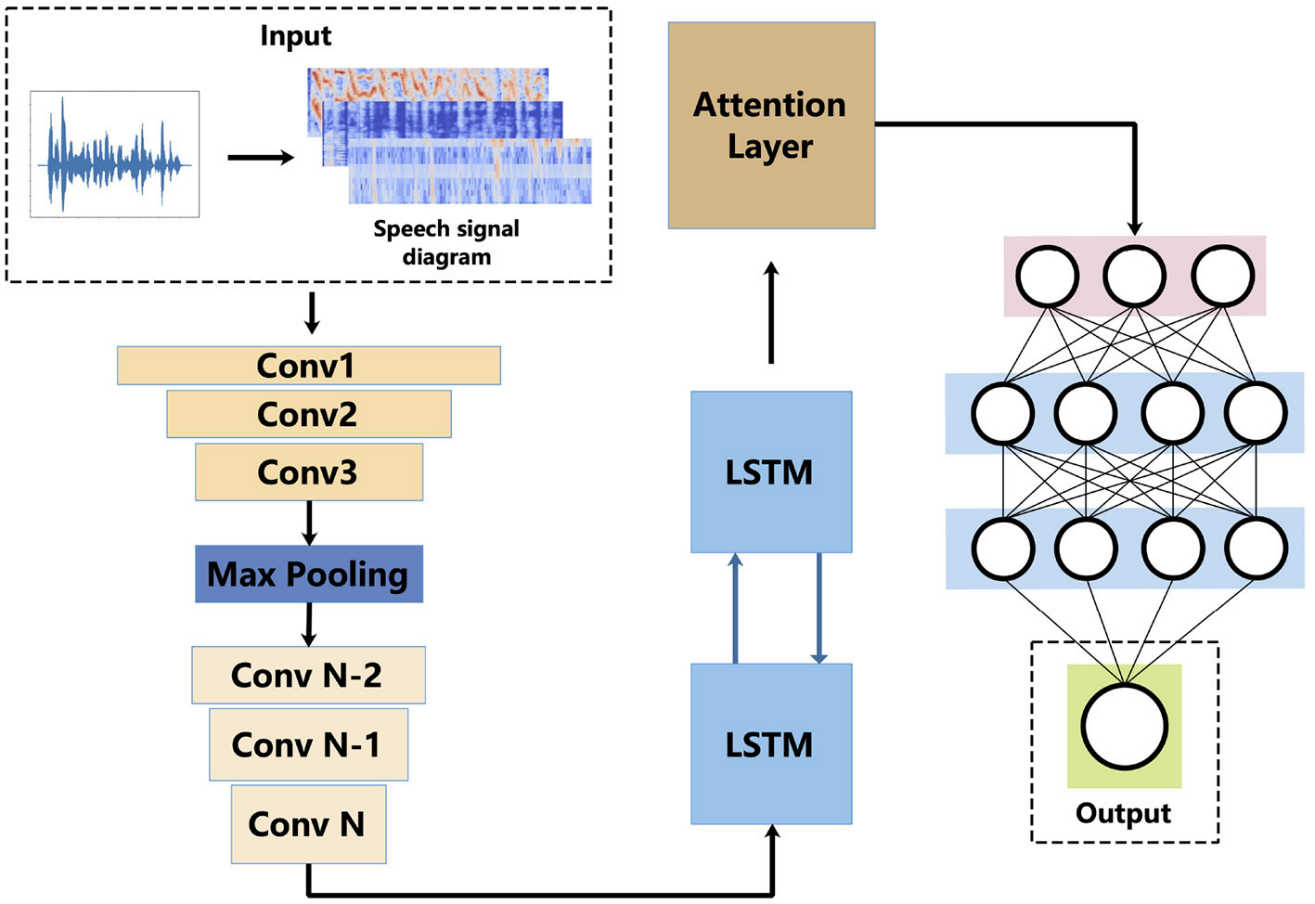
Architecture of ResCNN-SER. This figure shows the work flow of the ResCNN SER model. Firstly, log-Mels spectra (static, delta and delta) is computed. The log-Mels spectral properties can be seen as a kind of image. On this basis, a convolutional neural network (CNN) is used for feature extraction. Using the LSTM model to extract features from time serious data. We use the attention-based mode to reinforce feature representation. A complete neural network to predict language emotion.

We developed a method named ResCNN-SER to predict emotions with the speech signal in our previous work. Figure 1 shows the structure of ResCNN-SER. The framework includes the following five steps: First, a log-Mels spectrogram (static, delta, and delta-deltas) is used to extract the feature of the input speech signal. Second, a convolutional neural network (CNN) with residual blocks is used to extract log-Mels features from data containing delta and delta-delta. The third step is to use LSTM (LSTM) to comprehensively consider sequence signal . Fourth, we employ an attention-based model to obtain the important features. On this basis, speech emotion is predicted using a complete connected layer.

#### 2.1.1. Feature extraction based on Llog-Mels spectrogram

Extracting features precisely from speech signal is a key factor affecting speech emotion recognition. A large number of existing studies have shown that converting speech data into LIog-Mers spectral-based representation can improve the effect of speech emotion recognition (Chen and Zhao, 2020; Zayene et al., 2020). Through Llog-Mels spectrogram, the difficulty of speech recognition due to differences in style and intonation can be reduced (Chen et al., 2018).

In ResCNN-SER, a logic-Myers spectrum analysis is performed on a series of speech signals. In particular, in a sequence of speech signals, the z-score normalization algorithm is applied to normalize the voice signals. The sound signals are divided into several window frames. The size of the windows is set to 25 ms and the stride is set to 10 ms. For each window, Discrete Fourier Transform (DFT) is used to compute the spectrum. Then, we input the power spectrum of window *j* to the LIogMel-filter to obtain *q*_*j*_. Next, we perform the following logarithmic operations on *q*_*i*_:


(1) $$\begin{array}{l} f_{j}=\log\left(p_{j}\right) \end{array}$$

Then, we compute the deltas property of *f*_*j*_. In the case of *f*_*j*_, the delta characteristic $f_{j}^{d}$ is calculated as follows:


(2) $$\begin{array}{l} f_{j}^{d}=\frac{\sum_{n=1}^{N}n(f_{j+n}-f_{j-n})}{2\sum_{n=1}^{N}n^{2}} \end{array}$$

Where *j*, *d* represent the number of windows and corresponding deltas. Based on Keren and Schuller (2016). At last, we calculate deltas-deltas $f_{j}^{dd}$ as follows:


(3) $$\begin{array}{l} f_{j}^{dd}=\frac{\sum_{n=1}^{N}n(f_{j+n}^{d}-f_{j+n}^{d})}{2\sum_{n=1}^{N}n^{2}} \end{array}$$

After obtaining the *f*_*j*_, $f_{j}^{d}$ and $f_{j}^{dd}$, the three features are combined. A three-dimension feature *F*∈ℝ^t*k*c^ of the input voice signal is constructed. *t* is the size of time window. *k* is the size of LIogMel-filter bank. *c* is the size of feature. Here, c is equal to three as described previously.

#### 2.1.2. CNN-based speech feature representation

Convolutional neural networks have good application prospects in image analysis, text mining, and biomedical data analysis. On this basis, a series of multiple convolutional layer models are established. However, when the number of layers of the model is too many, the feature information will be lost during the information transfer. Currently, research on computer vision and natural language processing techniques has been proposed. For instance, Residual structure (ResNet) is proposed to avoid information loss in the deep convolutional neural networks (He et al., 2016). Based on the ResNet model, we also use a residual block-based neural network to avoid the information loss. In the ResCNN-SER system, CNN and residual block model are designed for feature representation of voice data. Given a three-dimension feature obtained from previous step, in order to use the CNN and residual block-based model, we applied 128 kernels to enhance the feature information. Second, feature extraction is performed using two convolutional layers. Every convolutional layer in this step also has 128 kernels and use residual blocks to prevent loss of information. We combine outputs of the first convolution with the output of the third convolution. Then, we use the max pooling layer to reduce the dimension of the data. Similar to what was done before, we utilize multiple convolutional layers and residual blocks for feature extraction of speech.

#### 2.1.3. Bi-LSTM-based feature representation

LSTM (Hochreiter and Schmidhuber, 1997; Gers et al., 1999) is a modeling method based on RNN. In text mining research area, LSTM is usually applied to obtain context knowledge of words or sentences (Wang et al., 2016). LSTM may obtain the distant association among data points in the time series or sequencial data. Here, a bi-direction LSTM (BiLSTM)-based model (Graves and Schmidhuber, 2005; Huang et al., 2015) is applied for feature representation. The BiLSTM-based model consists of two parts: forward part and reverse part, which are designed for feature representation for both directions.

The building block of LSTM is also neural network, containing forget gate, input gate and output gate. In the time serious data or sequential data, at data point *t*, the output of a layer could be associated with the previous layer or the next layer. For the rest of the previous and present information, forget and input gate are used, respectively. Combine the result of these two gates, we make the output gate produce the hidden features.

#### 2.1.4. Attention-based model for the feature extraction

In this part, we use an attention-based model to strengthen the feature representation ability of our method. Attention-based model has been very successfully used in many areas, such as text mining (Yin et al., 2016; Vaswani et al., 2017). On this basis, we introduce the attention method into the our method.

In a Bi-LSTM block, $h_{t}=\left[\overset{⃖}{h_{i}},\vec{h_{i}}\right]$, *h*_*i*_ is the feature at layer *i* in two directions. Let α_*i*_ be the weight of *h*_*i*_. Mathematically, the softmax is applied to compute the α_*i*_:


(4) $$\begin{array}{l} \alpha_{i}=\frac{e^{\sigma (W_{a}*h_{i}+b)*W_{b}}}{\sum_{i=1}^{T}e^{\sigma (W_{a}*h_{i}+b)*W_{b}}} \end{array}$$

In this equation, σ represents the sigmoid function, *W*_*a*_, *W*_*b*_ and *b* are parameters to be trained. We can compute the representation *r* based on the weight α_*i*_:


(5) $$\begin{array}{l} r=\overset{T}{\underset{i=1}{\sum }}\alpha_{i}h_{i} \end{array}$$

#### 2.1.5. Speech emotion prediction based on neural network

Following the attention-based part, a fully connected neural network is employed to recognize emotion based on voice signals. We employ a two-level neural network for prediction. In the last layer, we can use the softmax function to obtain a predicted label *p*_*ic*_. ResCNN-SER is then trained with cross-entropy loss.

### 2.2. The influence of the sound environment at court on the defendant's emotion

This paper explores how the courtroom sound environment affects the defendant's emotions from three aspects. First, we construct a data set for the research purpose. Second, for model training, we manually annotated the dataset. At last, we trained the ResCNN-SER based on the annotated dataset, and then used the trained model to other data to analyze the relationship between defendant's emotion and the judge and the plaintiff‘s speech.

#### 2.2.1. Dataset generation

Due to the lack of available data to analyze the impact of the court's acoustic environment on defendants' emotions, we produced 50 court hearing recordings at court from Datong People's Court, Daqing, Heilongjiang, China. These trial records are also public available at http://tingshen.court.gov.cn. The lengths of time for used cases range from 10 to 60 min. Two preprocessing stages are proposed. In stage one, we filtered the long stop between the two sentences of voice. The interval between two sentences of speech is set to 1 s. Then, in each record from the court, sentences are divided into three types: Judge, Plaintiff, and Defendant.

#### 2.2.2. Dataset annotation

Through the analysis of the emotional impact of the defendants, it is very necessary to identify the emotions of the people in court. But relying on professional experts to manually identify human emotions is time-consuming. So we use the proposed ResCNN-SER method to automatically predict people's emotions in courtroom speeches. On this basis, we use the ten speech transcripts with expert labels as training data to train the ResCNN-SER model. Three experts annotated each experimental record. In each sentence of record, if more than two experts labeled the same emotion, the sentensce is considered as the type of emotion. By training the ResCNN-SER, we can automatically analyze the data and predict emotions.

#### 2.2.3. Defendant's emotion analysis

The purpose of this study is to explore the impact of court sound environment on defendants' emotion. When analyzing it, the sound environment of the court includes the voices of judges and plaintiffs. Therefore, this paper mainly studies how the voice of the judge and the plaintiff affect the emotion of the defendant. More specifically, we will focus on analyzing whether the voice of the judge and the plaintiff has an impact on the defendant's emotion. In addition, this study attempts to explore the relationship between the emotion of the judge and the defendant when speaking to the plaintiff. We analyzed the defendant's emotion from three aspects. First, we use the ResCNN-SER model to determine the defendant's emotion in each record. Although there are some elements about the judge and plaintiff that can affect the defendant's emotion, our main concern is the affect of the voice. So, in step 2, we utilize ResCNN-SER to determine the emotions of judges and plaintiffs. Finally, we evaluate the defendant's emotion for the effect of the auditory environment.

## 3. Results and discussion

### 3.1. Influence of judge's voice at court on defendant's emotion

Using the ResCNN-SER model, we are able to identify people's emotions in court. By analyzing the 50 case recordings, the impact of the court's acoustic environment was analyzed. In this investigation, the voices of the judge and the plaintiff were considered the acoustic environment.

**Figure 2:**
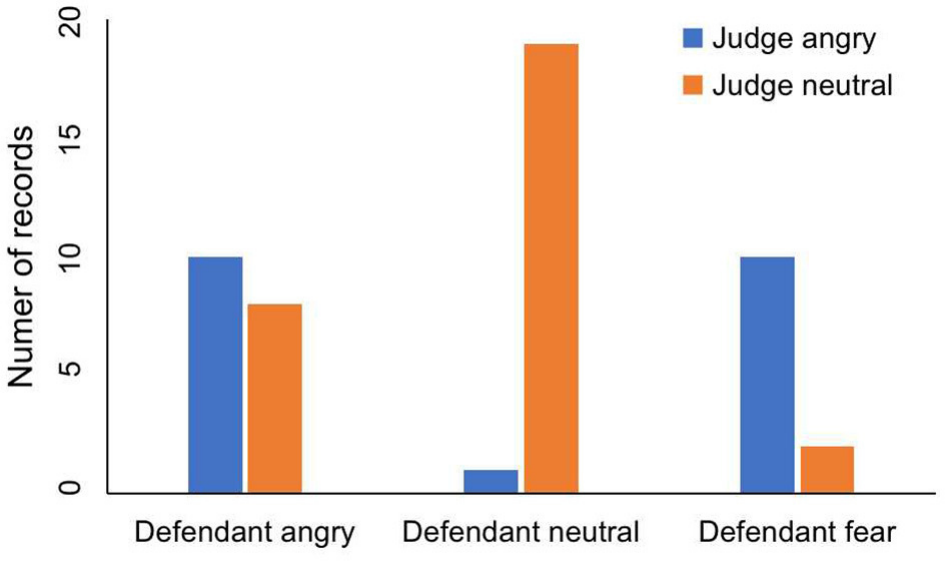
The influence of judge's emotion on defendant's emotion.

The emotions are divided into three categories: anger, neutrality and fear. The judge's tone has two emotions. One is angry and the other is neutral. In Table 1 and Figure 2, we can see the judge's emotion and the defendant's emotion. Ten records show that the judge was angry and the defendant was afraid. Nineteen records show that the defendant was neutral while the judge remained neutral. However, only one record shows that the defendant was in a neutral state when the judge was angry. This result suggests that the judge's anger may make the defendant be not netral. Regardless of whether the judge was angry or neutral, the numbers of records in which defendant is angry are ten and eight. This shows that the defendant's anger has nothing to do with the judge's emotion.

**Table 1:** The influence of judge's emotion on defendant's emotion.

	**Defendant angry**	**Defendant neutral**	**Defendant fear**
Judge angry	10	1	10
Judge neutral	8	19	2

The columns are the defendant's emotions. The rows are the judge's emotions. The numbers are the number of trial records in this type of conditions.

### 3.2. Influence of plaintiff's voice at court on defendant's emotion

**Figure 3:**
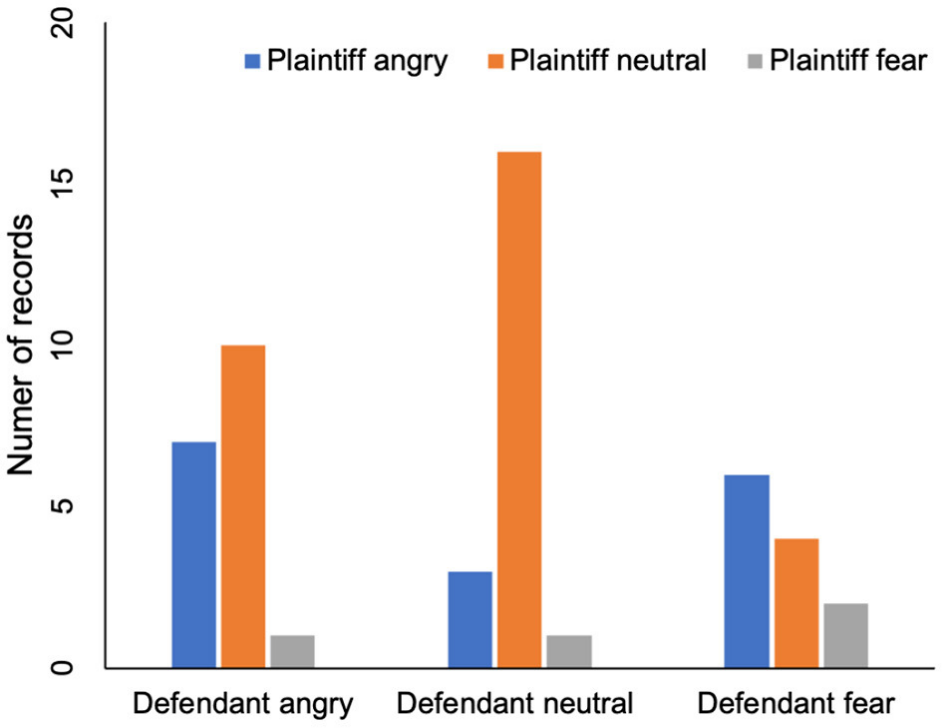
The influence of plaintiff's emotion on defendant's emotion.

In addition, this paper also analyzes the role of the plaintiff's voice in the trial. Again, we need to understand the plaintiff's and defendant's emotions first. The plaintiff's voice was divided into three categories: angry, neutral and fearful. The plaintiff's emotion and the defendant's emotion are shown in Table 2 and Figure 3. In six cases, when the plaintiff was angry, the defendant was afraid. On four records, the defendants were frightened by the plaintiff's neutrality.This indicates that defendant's fear emotion may not be affected by the plaintiff's emotion. Table 2 also shows that the number of records ,in which defendant is angry when plaintiff is angry or neutral, are seven and ten, respectively. There is no significant difference, which indicates that defendant's angry emotion is not affected by plaintiff's emotion either.

**Table 2:** The influence of plaintiff's emotion on defendant's emotion.

	**Defendant angry**	**Defendant neutral**	**Defendant fear**
Plaintiff angry	7	3	6
Plaintiff neutral	10	16	4
Plaintiff fear	1	1	2

The columns are the defendant's emotions. The rows are the judge's emotions. The numbers are the number of trail records in this type of conditions.

### 3.3. Influence of voice at court on defendant's logic

**Figure 4:**
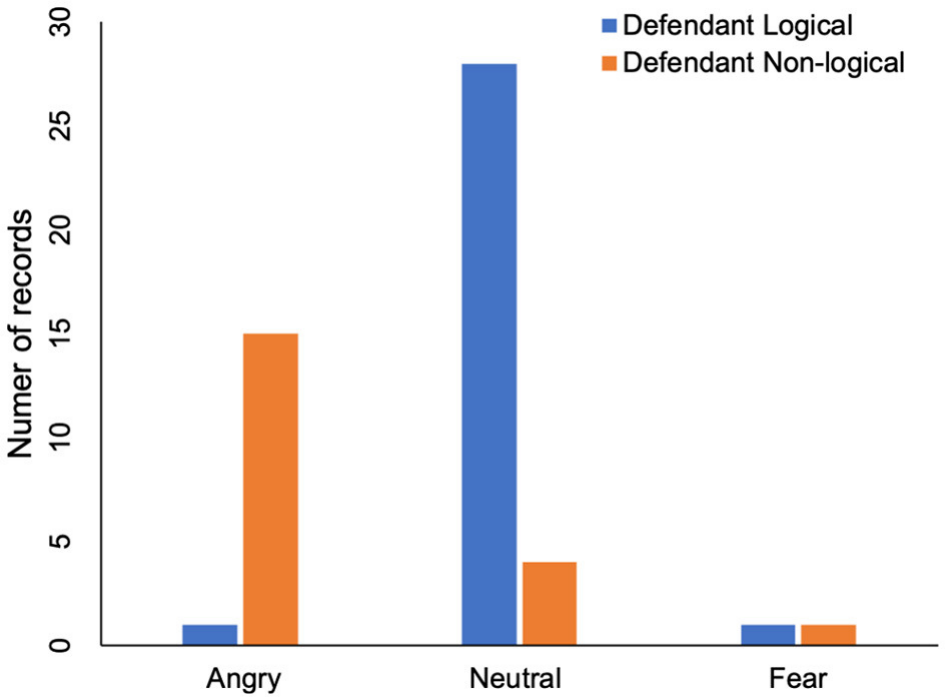
The influence of voice at court on defendant's logic.

Furthermore, we analyze how the voice affect the defendant's logic at court. It can be the voice of the judge, the plaintiff or the public prosecutor. We first recognize the voice and defendant's logic. Three types of emotions are predicted from voice at court, which are angry, neutral and fear. The relation between court's voice and defendant's logic is shown in Table 3 and Figure 4. In one record, the defendant's logic is clear, and in 15 records, the defendant's logic is not clear when the emotion at court is angry. This indicates that defendant's logic may be affected by the angry voice. In 28 records, the defendant's logic is clear, and in four records, the defendant's logic is not clear when the emotion at court is neutral.This also indicates that defendant's logic may be affected by the voice at court. Table 3 also shows that the number of records when the court's voice environment is fear. The result shows that there is no significant difference, which indicates that defendant's logic is not affected by the fear emotion.

**Table 3:** The influence of voice at court on defendant's logic.

	**Angry**	**Neutral**	**Fear**
Defendant logical	1	28	1
Defendant non-logical	15	4	1

The columns are the emotions of people at court. The rows are the defendant's logic. The numbers are the number of trail records in this type of conditions.

## 4. Conclusion

In this paper, we used a speech-based emotions prediction method, termed as ResCNN-SER, for predicting people's emotions at court. The method utilizes multiple neural network components to recognize speech signals and perform emotion prediction. We first take the sound signal as input and then calculate the log-Mels spectrogram (static, delta and delta-delta). Secondly, using the Log-Mels spectral characteristics, the speech signal is represented by using convolutional neural network, short-term memory and attention-based model. Finally, a fully connected neural network is applied for emotion classification. Using ResCNN-SER for emotion prediction, we provide an analysis of how the voice of the court affects defendants. The results showed that when the judge was angry, the defendant was afraid. Plaintiff's emotion has little effect on defendant's emotion.

## Data availability statement

The raw data supporting the conclusions of this article will be made available by the authors, without undue reservation.

## Author contributions

YS conceived the idea and design the project and wrote the manuscript. TZ performs the analysis and revised the manuscript. All authors contributed to the article and approved the submitted version.
